# Toxicological evaluation of water-extract sericin from silkworm (*Bombyx mori*) in pregnant rats and their fetus during pregnancy

**DOI:** 10.3389/fphar.2022.982841

**Published:** 2022-09-02

**Authors:** Jinyue Li, Pingjing Wen, Guangqiu Qin, Jiehong Zhang, Peng Zhao, Yixin Ye

**Affiliations:** ^1^ Department of Preventive Medicine, Guangxi University of Chinese Medicine, Nanning, China; ^2^ Institute of Toxicology, Guangxi Center for Disease Prevention and Control, Nanning, China

**Keywords:** sericin, teratogenicity, repeated oral administration, safety assessment, oral toxicity in rats

## Abstract

Sericin is a natural protein produced by the silkworm *Bombyx mori*, which has a wide range of biological activities and has a broad application prospect in multiple areas. However, systemic toxicity and safety assessment of sericin is still rare. This study was aimed to evaluate the toxic effects of water-extract sericin from cocoons of *Bombyx mori* in pregnant rats and their fetuses during pregnancy. Eighty pregnant rats were randomly divided into three treatment groups, one negative and one positive control group. The treatment groups were administered water-extract sericin solutions at doses of 1,000, 500, and 250 mg/kg, while the negative and positive control groups were administered pure water and 300 mg/kg aspirin, respectively. Rats were exposed daily by oral gavage from the seventh day of gestation for 10 consecutive days and sacrificed on the 20th day of gestation. The results showed that water-extract sericin did not induce any treatment-related changes on pregnant rats (clinical signs, body weights, food consumption, ovarian and uterine weights) and fetuses (body weights, body lengths, tail lengths, visceral, and skeletal development). The no-observed-adverse-effect-level (NOAEL) of sericin was determined to be 1,000 mg/kg body weight in rats. These results indicated that water-extract sericin is of low teratogenic potential under the experimental conditions of this study.

## Introduction

Sericin is a natural protein produced by the silkworm *Bombyx mori*, that acts like a gum holding the fibroin fibers together for reconstruction of the cocoon shells and accounts for 20–30% of the total cocoon weight (Kundu et al., 2008; Wang et al., 2012; Cao and Zhang, 2016). Sericin contains 18 amino acids with eight essential amino acids (Lysine, phenylalanine, methionine, threonine, isoleucine, leucine, valine, histidine) that play important roles in human metabolic pathways (Kunz et al., 2016). Traditionally, sericin is extracted by a hot water degumming process during commercial silk production and then discarded (Cao and Zhang, 2016).

Silkworm cocoons and the related ingredients have long been used in traditional medicine. In East Asia, cooked water of silkworm cocoons (mainly sericin) has been widely used to treat diabetes and hypertension. In the Unani system of medicine practiced in south Asia and the Middle East, sericin is extensively used as the main ingredient of various polyherbal formulations in cardiac and neurological related diseases (Khan et al., 2006; Ahsan et al., 2021). With the development of modern medicine, it is found that sericin has rich protein structure and amino acid groups with multiple biological and pharmacological activities, including antioxidant (Kumar and Mandal, 2017), antitumor (Sasaki et al., 2000), anti-inflammatory (Aramwit et al., 2013), anticoagulation, hypoglycemic and cholesterol-lowering activities (Kunz et al., 2016; Dong et al., 2020). Moreover, the easily cross-linked and copolymerized nature of the sericin structure offers an unlimited possibility for its combination with other molecular materials into new high-end materials. For example, when associated engineered nanoparticles, sericin-based products showed significant antimicrobial and wound healing activities that could be applied to wound care (Gilotra et al., 2018; Shah et al., 2019; Muhammad Tahir et al., 2020). Another study found sericin a promising material in tissue engineering and in drug delivery as a culture medium and cryopreservation (Chithrashree et al., 2021). Based on its biological activities and unique biochemical features, sericin has attracted much attentions as an additive in the food, cosmetic, medical and pharmaceutical industries (Cao and Zhang, 2016).

While the functional properties of sericin and have been extensively studied, the biosafety of sericin remains controversial. Previously, sericin was associated with childhood asthma, allergenicity, immunogenicity and cytotoxicity (Celedón et al., 2001; Thitiwuthikiat et al., 2010; Jiao et al., 2017), while other research claimed that sericin exhibits mild inflammatory responses, negligible allergenicity, and low immunogenicity *in vivo* (Jiao et al., 2017). To evaluate whether intake of sericin induces undesired toxic effects, we conducted a battery of *in vivo* and *in vitro* studies to examine the toxicity of water-extract sericin. Our previous studies showed that water-extract sericin was low genotoxicity and subchronic toxicity (Qin et al., 2020). As part of a comprehensive toxicological assessment, this study was aimed to evaluate the potential toxicity of sericin to pregnant rats and their fetus when administered daily by gavage to pregnant rats, so as to provide experimental evidence for safety assessment of sericin-related products *in vivo*.

## Methods and materials

### Sericin extraction and its characterization

Fresh *Bombyx mori* cocoons were provided by the Guangxi Institute for Product Quality Inspection (Nanning, China). Sericin extract used in this study was the same batch used in previous study (Qin et al., 2020). Extraction of sericin from cocoons and its characterization was described previously (Aramwit et al., 2010; Qin et al., 2020). Briefly, cocoons without pupa were cut into pieces and degummed in deionized water at a ratio of 1:30 (w: v) at 100°C for 3 h twice. The aqueous solution was mixed, filtered to remove insoluble components and freeze-dried to obtain sericin powder. Amino acids components of the sericin extract were analyzed with an amino acid analyzer (Hitachi L-8500A, Tokyo, Japan).

### Experimental animals

A total of 150 specific pathogen-free adult sexually mature unmated Sprague-Dawley (SD) rats (50 males and 100 females) were provided by the Animal Experimental Center at Guangdong Academy of Medical Science (Guangzhou, China). Rats were kept in the Experimental Animal Center of Guangxi Center for Disease Prevention and Control (Nanning, China), in a constant temperature and humidity barrier system with a temperature of 22–25°C and a relative humidity of 55–70%. The indoor lighting was provided for 12 h a day. Animals were quarantined for 7 days before the experiment, provided with conventional diets and sterilized tap water. Diets were provided by the Animal Experimental Center at Guangdong Academy of Medical Science (Guangzhou, China). The composition of the diets includes 40% corn powder, 20% soybean meal, 23% flour, 7% fish meal, 2.2% calcium hydrogen phosphate, 3.5% yeast powder, 1.4% calcium carbonate, 1% mixed vitamins, 1.5% corn oil and 0.4% salt. Protocols of animal study were approved by the Animal Experimentation Ethics Committee at Guangxi Center for Disease Prevention and Control (No. 20170009).

### Animal study

Animal study was conducted according to the guidelines from the National Food Safety Standard of China for Teratogenic Test (NHFPCC, 2015), China National Accreditation Service for Conformity Assessment (CNAS-RL01) and China Metrology Accreditation.

In this study, 100 female and 50 male rats were used for mating. Every two female rats were housed with one male rat overnight for mating in a polycarbonate cage. Successful mating was confirmed by vaginal smear examining the presence of vaginal plugs or sperms on the next morning. The day of checking was considered day 0 of gestation. Mated females were separated in cages individually, and provided with conventional diets and sterilized tap water.

On gestation day (GD) 7, pregnant rats were weighed and randomly assigned into one negative control, one positive control group, and three treatment groups, with 16 rats in each group. The treatment groups were given 1,000, 500, and 250 mg/kg body weight sericin extract, respectively. The negative control group was given pure water, while the positive control group was given 300 mg/kg aspirin (30 mg/ml aspirin solution at a dosing volume of 10 ml/kg body weight). Rats were gavaged once a day same time in the early morning for 10 consecutive days until the 16th day of pregnancy.

The treatment doses were selected based on results from our previous 90-days subchronic toxicity study (Qin et al., 2020). In the subchronic toxicity study, the NOAEL of sericin was observed to be 1,000 mg/kg in SD rats by gavage. The positive control and its dose (300 mg/kg aspirin) was selected following the suggestion of the National Food Safety Standard for Teratogenic Test (NHFPCC, 2015). Although it was reported that low doses of aspirin are effective in prevention of preeclampsia in high-risk patients (Atallah et al., 2017), high doses of aspirin were reported to induce substantial reproductive and developmental toxicity in laboratory animals (Okamoto et al., 1988; Zhao 2010).

Throughout the study, general signs of toxicity and mortality was monitored daily, including physical signs, appearance, behavior, urine, feces and fur. Food consumption was recorded twice a week. Body weights of rats was recorded on gestation days 0, 7, 12, 16, and 20. Values of food intake and body weights were present as the mean of the intermediate days. On the 20th day of pregnancy, all pregnant rats were sacrificed after anesthesia with pentobarbital sodium. In the meantime, a gross autopsy of each rat was conducted. The ovaries and the uterus were removed and weighed. Each fetus was removed from the uterus to identify its sex. The body lengths, tail lengths and weights of fetuses were recorded. Luteal and fetal status of all female rats were observed and recorded. Specimens of liver, spleen, kidneys and ovaries from pregnant rats were collected and fixed in 4% neutral buffered formaldehyde, embedded in paraffin, stained with Giesma, and then examined under a Leica DM 6000B microscopy (Wetzler, Germany). The degree of histopathological lesions was scored according to the severity of the lesions.

After the above examination, fetuses from each litter were randomly divided into two groups for skeletal and visceral examination, respectively. For skeletal examination, fetuses were immobilized with 95% (v/v) ethanol for 2 weeks, cleared with 1.5% (w/w) KOH for 2 days, and stained with Alizarin Red S for 2 days before observation under an optical microscope. For visceral examination, fetuses were immobilized in Bouin’s fluid for 2 weeks before examination.

### Statistical analysis

SPSS version 21.0 (SPSS Inc, Chicago, Illinois, United States) was used for statistical processing of the experimental data. Quantitative variables were present as mean ± standard deviations. Homogeneity of variances was examined by Bartlett’s test. Difference between quantitative variables was compared using a one-way ANOVA followed by Dunnett’s test, while difference between categorized variables was compared by Pearson’s Chi-square test. A *p*-value less than 0.05 was considered significant.

## Results

### Composition of amino acids in sericin

As reported previously, 17 amino acids were detected in the sericin extract. The most abundant amino acid components in the sericin were serine (26.0%), aspartic acid (16.5%), and threonine (9.8%) (Qin et al., 2020).

### General clinical signs of animals

No abnormal clinical signs (appearance, behavior, urine, feces and fur) were observed in rats of the treatment groups. No mortality was recorded.

### Body weights

**FIGURE 1:**
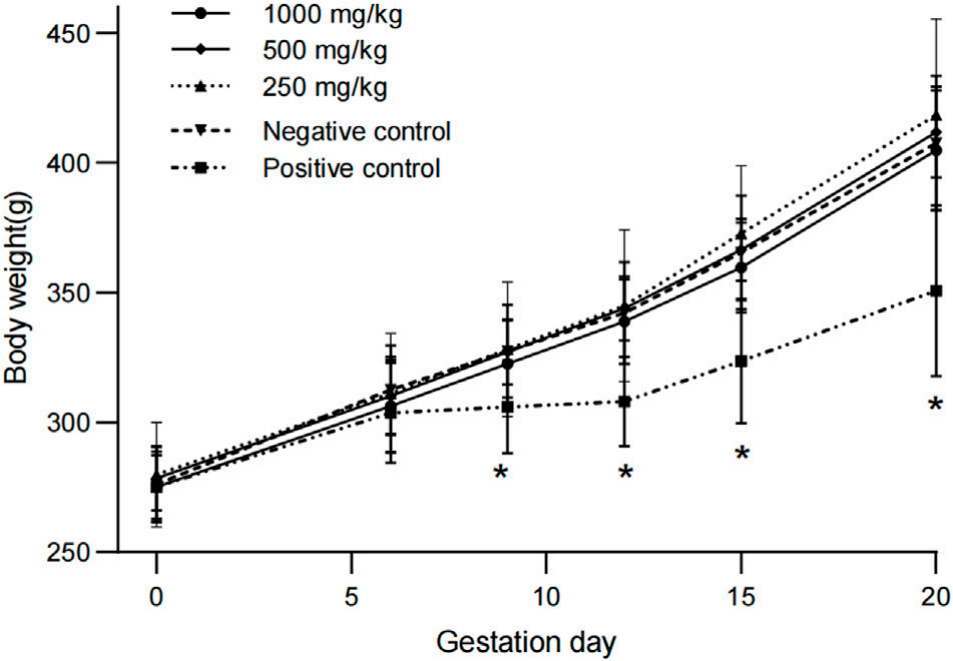
Weight changes of pregnant rats treated with water-extract sericin. Asterisks (*) indicate significant difference compared to the negative control (*p* < 0.05).

Compared with the negative control group, the pregnancy weights and weight gains of rats in all dose groups were similar. The body weights and weight gains of pregnant rats in positive control group were significantly lower than those in negative control group at day 9, 12, and 20 (*p* < 0.05, Figure 1). There were no treatment-related changes in food consumption rates and food utilization rates in rats treated with sericin (data not shown).

### Maternal examination

**FIGURE 2:**
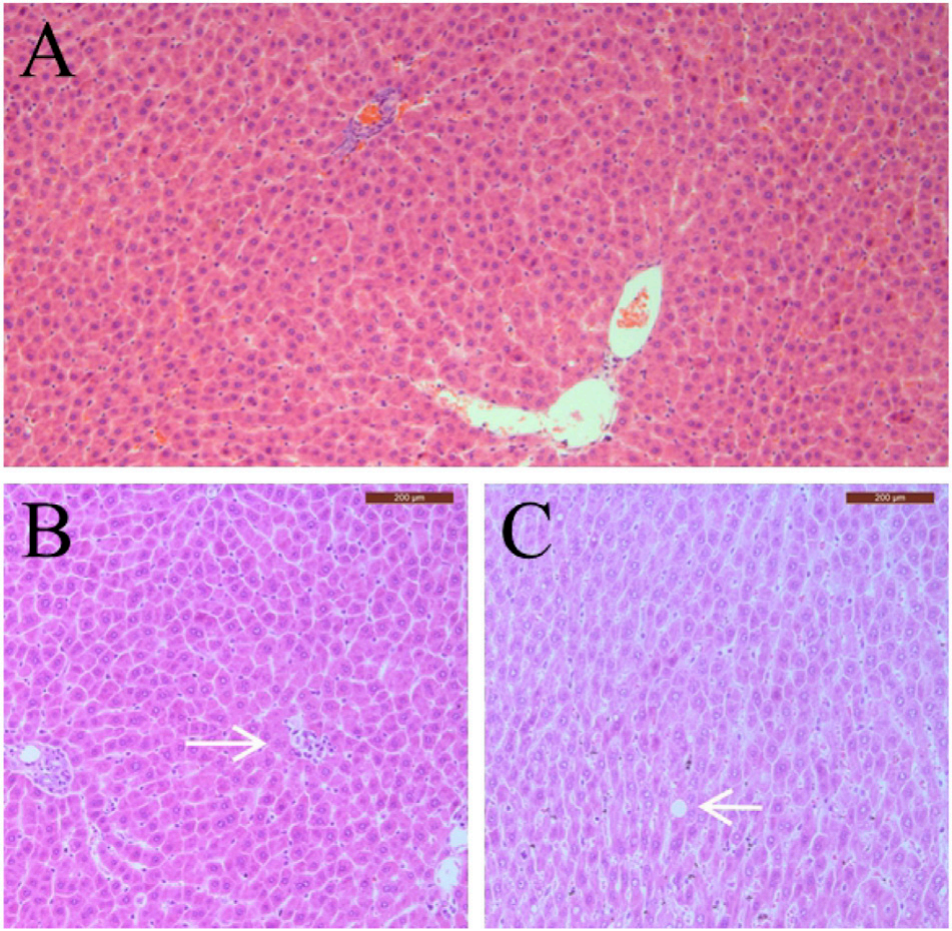
Typical pathological changes observed in pregnant rats in this study. **(A)** Normal hepatocytes; **(B)** Spotty necrosis of hepatocytes; **(C)** Fatty degeneration of hepatocytes. Arrow indicates pathological changes.

Complete gross necropsy was conducted on all pregnant rats and no treatment-related change was observed in rats of the treatment groups. Therefore, histopathological examinations were conducted only on the 1,000 mg/kg sericin treatment group and the negative control group. For the treatment group, mild spotty necrosis of hepatocytes was observed in one rat and mild fatty degeneration of hepatocytes was observed in two rats (Figure 2). Same types and similar severity of histopathological lesions were observed in two rats of the negative control group.

A total of 206 implantations were observed in the negative control, with 204 live fetuses, one dead fetus and one resorption. There was no statistical significance between rats in treatment groups and control group regarding the average gravid uterus weights, number of live fetuses per litter and number of implantations and resorptions (*p* > 0.05; Table 1). However, significant difference was observed between positive control group and negative control group, on gravid uterus weights, ratios of live and dead fetuses, number of live fetuses per liter and resorption rates (*p* < 0.01; Table 1).

**TABLE 1:** Effects of water-extract sericin on reproductive parameters of rats.

Dose (mg/kg)	Number of litters	Total implantation number	Gravid uterus weights (g)			Dead fetuses		Live fetuses		Number of live fetuses per litter			Resorption	
						Number	Ratio (%)	Number	Ratio (%)				Number	Ratio (%)
1,000	16	204	72.6	±	12.5	2	0.98	198	97.06	12.4	±	2.0	4	1.96
500	16	222	80.1	±	11.2	1	0.45	217	97.75	13.6	±	1.9	4	1.80
250	16	222	83.1	±	19.5	1	0.45	216	97.30	13.5	±	3.2	5	2.25
Negative control	16	206	76.9	±	23.9	1	0.49	204	99.03	12.7	±	4.0	1	0.49
Positive control	16	178	29.6	±	20.2**	25	14.04^**^	74	41.57^**^	6.1	±	4.8^**^	79	45.38^**^

** indicates *p* < 0.01 compared with the negative control group.

### Fetal examination

The average body weights, body lengths and tail lengths of fetuses in the negative control were 3.59 g, 37.26, and 13.69 mm, respectively. The body weights, body lengths and tail lengths of fetuses in treatment groups were similar to those of the negative control group (*p* > 0.05); while body weights, body lengths and tail lengths of fetuses in the positive control group were significantly lower than those in the negative control group (*p <* 0.05, Table 2).

**TABLE 2:** Effect of water-extract sericin on growth and development of fetuses.

Dose (mg/kg)	Number of fetuses examined	Body weights (g)	Body lengths (mm)	Tail lengths (mm)
1,000	198	3.60 ± 0.26	37.68 ± 1.13	13.67 ± 0.43
500	217	3.63 ± 0.20	37.50 ± 1.01	13.86 ± 0.43
250	216	3.63 ± 0.25	37.61 ± 1.28	13.74 ± 0.47
Negative control	204	3.59 ± 0.28	37.26 ± 1.54	13.69 ± 0.57
Positive control	74	2.63 ± 0.19**	32.80 ± 2.47**	12.86 ± 0.50**

** indicates *p* < 0.01 compared with the negative control group.

**FIGURE 3:**
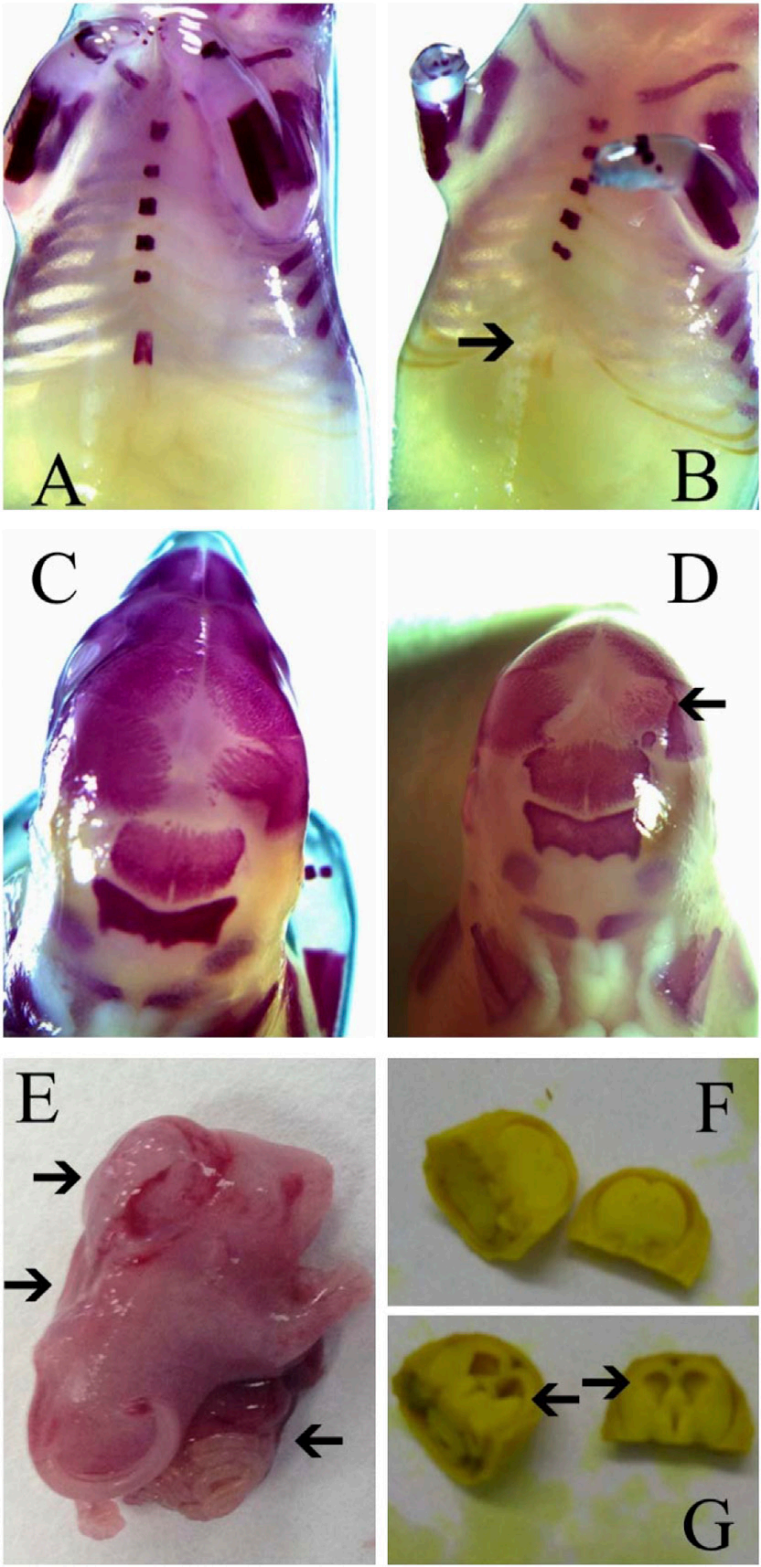
Typical malformations observed in fetuses of rats in this study. **(A)** Normal sternum; **(B)** Sternal defect; **(C)** Normal skull; **(D)** Skull ossification retardation; **(E)** Encephalocele, spina bifida and abdominal fissure; **(F)** Normal lateral ventricle; **(G)** Lateral ventricle enlargement. Arrow indicates malformations.

For the external examination, a total of 630 fetuses in the treatment groups and 204 fetuses in the negative control group were examined. No abnormality was observed in the external appearance of these fetuses. In the positive control group, 74 fetuses were examined and 14 fetuses with external malformations were found. These external malformations included 12 cases of encephalocele, 9 cases of abdominal fissure and 10 cases of spina bifida (Figure 3 and Table 3).

**TABLE 3:** Effects of water-extract sericin on external malformations of fetuses.

Dose (mg/kg)	Number of fetuses examined	Number of fetuses with external malformations	Abnormal tail	Limb valgus	Encephalocele	Abdominal fissure	Umbilical hernia	Spina bifida
1,000	198	0	0	0	0	0	0	0
500	217	0	0	0	0	0	0	0
250	216	0	0	0	0	0	0	0
Negative control	204	0	0	0	0	0	0	0
Positive control	74	14**	0	0	12**	9**	0	10**

** indicates *p* < 0.01 compared with the negative control group.

For the visceral examination, a total of 459 fetuses were examined. No visceral malformation was observed in fetuses of the treatment and negative control groups, while 3 cases of lateral ventricle enlargement were observed in the positive control group (Figure 3 and Table 4).

**TABLE 4:** Effects of water-extract sericin on visceral development of fetuses.

Dose (mg/kg)	Number of fetuses examined	Visceral alterations	Lateral ventricle enlargement	Cleft palate	Abdominal kidney
1,000	98	0	0	0	0
500	110	0	0	0	0
250	110	0	0	0	0
Negative control	103	0	0	0	0
Positive control	38	3**	3**	0	0

** indicates *p* < 0.01 compared with the negative control group.

For skeletal examination, it was found that frontal suture widths of fetuses in the positive control group increased significantly compared with those of the negative control group (*p* < 0.01), while widths in the treatment groups was similar with those of the negative control (*p* > 0.05, Table 5). Sternal absences were found in all groups (Table 5; Figure 3). The ratios of sternal defects in the treatment groups were between 9.35% and 12.26, which were comparable to that of the negative control (12.87%, *p* > 0.05), while ratios of sternal defects in the positive control (55.55%) was significantly higher than that of the negative control (*p* < 0.01, Table 5).

**TABLE 5:** Effects of water-extract sericin on skeletal malformations of fetuses.

Dose (mg/kg)	Number of fetuses examined	Frontal suture width (mm)	Skull ossification retardation	Abnormal ribs	Abnormal spine	Abnormal tailbone	Sternal defect	Ratio of sternal defect (%)
1,000	100	1.91 ± 0.12	0	0	0	0	12	12.00
500	107	1.98 ± 0.20	0	0	0	0	10	9.35
250	106	1.94 ± 0.16	0	0	0	0	13	12.26
Negative control	101	1.93 ± 0.14	0	0	0	0	13	12.87
Positive control	36	2.43 ± 0.27**	5	0	0	3	20	55.55**

** indicates *p* < 0.01 compared with the negative control group.

## Discussion

Although increasing attention has been paid to sericin, research has been focused mainly to the processing and application (Ahsan et al., 2018), while knowledge regarding its systemic toxicity and safety assessment on purified sericin is limited. Previously, toxic effects of sericin and related products have been reported, including inflammation-inducing effects (Liu et al., 2007), allergenicity (Celedón et al., 2001) and cytotoxicity (Aramwit et al., 2020). For example, a clinical study reported severe inflammation reactions to virgin silk sutures (sericin-containing) in patients under cataract surgery, while silk sutures without sericin induced less inflammation reactions (Soong and Kenyon 1984). Another study reported that sericin-containing silk induced more severe inflammatory responses than sericin-free fibers to rats suffering subcutaneous implantation (Liu et al., 2007). However, other studies claimed that the *in vivo* inflammatory reactions caused by pure sericin were rather low and the observed inflammatory reactions might be due to contaminants in sericin, such as lipopolysaccharide and fibroin (Ersel et al., 2016; Jiao et al., 2017). A latest study on the absorption of sericin *in vivo* showed that sericin administered by oral gavage could be detected in serum of mice, and sericin with smaller molecular weight was more easily absorbed, indicating that at least some sericin can directly cross the gastrointestinal barrier and enter the blood circulation (Zhong 2022). However, the inflammatory response of sericin by oral ingestion has not been reported yet. Meanwhile, it was found that after a 3 h of simulated digestion *in vitro*, the molecular weight of sericin became smaller (from 25 to 260 kDa to 10–15 kDa) (Zhong 2022). These results indicate that sericin with large molecular weights may be digested into smaller molecular weights before absorption and consumption of sericin will likely not induce inflammation in humans. On the other hand, other studies claimed that sericin exhibits little toxicity (Jiao et al., 2017; Aramwit et al., 2020). It was reported that sericin-based hydrogel was well tolerable up to 3,800 mg/kg in rabbits (Al-Tabakha et al., 2021). Sericin-derived oligopeptides did not affect hematological parameters of BALB/c mice after 28-days exposure by gavage (Bunarsa et al., 2013).

Previously, we have conducted a battery of studies to evaluate the safety of water-extract sericin from *B. mori* cocoons, including three genotoxicity studies (the bacterial reverse mutation test, the mammalian erythrocyte micronucleus test and the mouse spermatogonia chromosomal aberration test) and a 90-days subchronic toxicity study in rats (Qin et al., 2020). Our previous results showed that water-extract sericin was non-mutagenic and non-genotoxic both *in vitro* and *in vivo*. Sericin was low toxicity in the sub-chronic toxicity study and the NOAEL was estimated to be 1 g/kg/day in SD rats. In another study assessing the acute and sub-acute toxicity of sericin, it was found that orally administered 2000 mg/kg sericin in a single dose did not induce any acute toxicity in mice, while repeatedly administered with 2000 mg/kg for 28 days induced inflammation in brain, small intestine and kidneys of mice (Ahsan et al., 2021). The NOAEL of sericin in mice was suggested to be below 2000 mg/kg in sub-acute study (Ahsan et al., 2021). These results indicate that there may be species difference in toxicity of sericin and long term high-dose ingestion of sericin may lead to toxic effects.

As part of a systemic toxicological assessment, the present study evaluated the potential teratogenic toxicity of water-extract sericin in rats. The experiments examined the effects of different doses of sericin on pregnant rats and their fetuses and compared with those of the control. Results of the study showed no treatment-related toxicity of sericin to pregnant rats. Body weights, food consumption, and food utilization rates of pregnant rats were not affected by administration of sericin at doses up to 1,000 mg/kg, comparing to the negative control. This result was consistent with previous study showing that 1,000 mg/kg sericin did not significantly influence body weights and food intakes of SD rats after a 90-days exposure by oral gavage (Qin et al., 2020). Another study reported that the body weights and white adipose tissue weights were unaffected by 4% dietary sericin in rats fed on a high-fat diet for 5 weeks, although reducing serum and hepatic lipids, improving glucose tolerance and elevating serum adiponectin concentration were observed (Okazaki et al., 2010). Meanwhile, another study reported that dietary feeding of sericin (0.38 and 1%) for 6 weeks significantly suppressed body weight gains and fat accumulation and improved the lipid metabolism in high fat-fed mice (Seo et al., 2011). Body weights of rats in the present study were not significant affected by administration of sericin. One possible reason is that sericin may be effective in suppressing body weight gains of high-fat diet animals while has little effects on animals fed with normal diet. Another reason may be that the exposure time needs to be long enough to induce the weight reduction effect. The antihyperlipidemic and body fat-lowering effects of sericin was considered to be partly due to the inhibition of hepatic and adipocyte lipogenesis and regulation of adipokine production in high-fat diet mice (Seo et al., 2011). A longer exposure period may facilitate the observation on the effect of sericin on body weight of animals.

Little attention has been paid to the potential reproductive and developmental toxicity of sericin. However, improvement effects of sericin were reported on post-thaw semen quality, oocyte fertilization and embryo development by supplementation with sericin *in vitro* (Isobe et al., 2012; Aghaz et al., 2015; Reddy et al., 2018). For example, Aghaz et al. reported that addition of 0.5% sericin improved the meiotic competence of oocytes and early embryonic development in Sanjabi ewes (Aghaz et al., 2015). The positive effect of sericin on reproductive cells and embryos was considered to be attributed to its antioxidative property (Isobe et al., 2012; Reddy et al., 2018). In the present study, reproductive and developmental effects of sericin was examined *in vivo*. The results showed that oral exposure of sericin did not affect number of implantations, indicating that oocyte fertilization of rats was not affected by sericin. Fetal number, growth and development was also not affected by treatment of sericin. Although sporadic sternum defects were observed in fetuses of all treatment groups, the number of cases and incidence rates were comparable to those in the negative control, thus were considered to be spontaneous malformations. The pathological changes in kidneys and liver of rats in the treatment group were minor and comparable to those of the negative control, therefore were considered to be without toxicological significance. This was consistent with result of the previous study showing that 1,000 mg/kg sericin did not induce significantly pathological changes in rats after exposed by gavage for 90 days (Qin et al., 2020). For the positive control group, it was found that 300 mg/kg aspirin significantly influenced body weights and gravid uterus weights of pregnant rats, and affected the number, growth and development of fetuses. Data for the positive and negative controls was within the range of historical control values in our lab. These results indicated that water-extract sericin has little teratogenic toxicity to SD rats under the experimental conditions of the current study. The NOAEL of sericin for the teratogenicity study in SD rats was determined to be 1,000 mg/kg.

One weak point of this study is the lack of blood hematology and serum biochemistry evaluation. Although it was reported in our previous study that blood parameters were not significantly affected by sericin of the same doses after 90-days continuous exposure in SD rats by gavage (Qin et al., 2020), blood indicators evaluation may better reveal the effects of sericin to pregnant rats and their fetuses. Moreover, pathological changes of fetuses are to be investigated in further studies.

## Conclusion

This study for the first time evaluated the toxicity of water-extract sericin from silkworm *B. mori* to pregnant rats and their fetuses. Results of the teratogenic toxicity study showed no treatment-related effects of sericin and the NOAEL was determined to be 1,000 mg/kg in SD rats.

## Data Availability

The raw data supporting the conclusions of this article will be made available by the authors, without undue reservation.
